# Spitzer shaped ZnO nanostructures for enhancement of field electron emission behaviors

**DOI:** 10.1039/c8ra03282c

**Published:** 2018-06-13

**Authors:** Parameshwar R. Chikate, Prashant K. Bankar, Ram J. Choudhary, Yuan-Ron Ma, Shankar I. Patil, Mahendra A. More, Deodatta M. Phase, Parasharam M. Shirage, Rupesh S. Devan

**Affiliations:** Discipline of Metallurgy Engineering and Materials Science, Indian Institute of Technology Indore Simrol Indore 453552 India rupesh@iiti.ac.in devan_rs@yahoo.co.in; Department of Physics, Savitribai Phule Pune University (Formerly, University of Pune) Pune 411007 India; UGC-DAE Consortium for Scientific Research Khandwa Road Indore 452001 India; Department of Physics, National Dong Hwa University Hualien 97401 Taiwan Republic of China

## Abstract

We observed enhanced field emission (FE) behavior for spitzer shaped ZnO nanowires synthesized *via* a hydrothermal approach. The spitzer shaped and pointed tipped 1D ZnO nanowires of average diameter 120 nm and length ∼5–6 μm were randomly grown over an ITO coated glass substrate. The turn-on field (*E*_on_) of 1.56 V μm^−1^ required to draw a current density of 10 μA cm^−2^ from these spitzer shaped ZnO nanowires is significantly lower than that of pristine and doped ZnO nanostructures, and MoS_2_@TiO_2_ heterostructure based FE devices. The orthodoxy test that was performed confirms the feasibility of a field enhancement factor (*β*_FE_) of 3924 for ZnO/ITO emitters. The enhancement in FE behavior can be attributed to the spitzer shaped nanotips, sharply pointed nanotips and individual dispersion of the ZnO nanowires. The ZnO/ITO emitters exhibited very stable electron emission with average current fluctuations of ±5%. Our investigations suggest that the spitzer shaped ZnO nanowires have potential for further improving in electron emission and other functionalities after forming tunable nano-hetero-architectures with metal or conducting materials.

## Abstract 2

Spitzer shaped ZnO nanowires had a reduced work function providing a significantly smaller barrier for the direct emission of an electron toward the emission site and contributed to a lowest turn-on field of 1.56 V μm^−1^.

## Graphical abstract

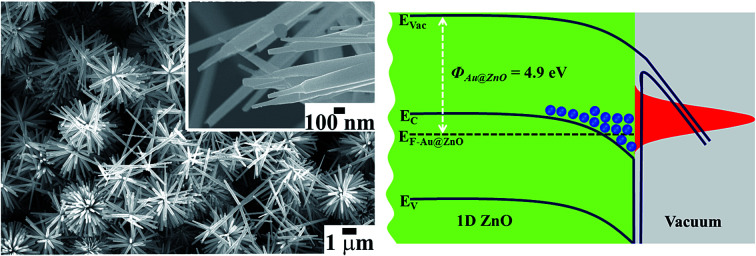

## Introduction

Among the various 1D nanostructure morphologies, nanowires and nanorods, offering the advantages of large surface areas, are found apposite to improve field electron emission. Carbon nanotubes are of great interest to field emission (FE) in particular because of their high aspect ratio, better electrical and thermal conductivity, and robust mechanical and chemical stability. However, the difficulties of establishing density controlled vertical nanotube growth at a lower cost have significantly impeded the practical execution of carbon nanotubes in field emission devices. Wide bandgap transition metal oxides such as NbO_2_, TiO_2_, CuO and SnO are known for their stability and are found to be suitable for field emission in their 1D forms such as wires, rods, tubes, needles *etc.*^1–4^ Even though ZnO is an attractive material for diverse applications in solar cells, catalysis, sensing, photocatalysis, smart windows, photoluminescence, supercapacitors, generators *etc.*, and is even more suitable for ultraviolet light emitters and laser diodes,^4,5^ it has only been moderately considered for use in FE displays because of its larger work function in the range of 5.3 to 5.6 eV, limited morphological forms and field screening effect from uncontrolled dispersion.^6–9^ Therefore, emerging approaches to tailor the work function and improve electron emission such as the modification of emitter geometry, the introduction of impurity, decoration of metals and the vertical alignment of the structures have been reported.^2,10^ The implantation of elements into ZnO nanowires was found to produce nanoscale protuberances and surface-related defects which reduced the turn-on field (*E*_on_) from 3.1 to 2.4 V μm^−1^ (at 0.1 μA cm^−2^).^11^ However, Cu doping in ZnO *via* direct current magnetron sputtering in an Ar and O_2_ environment deteriorated the crystalline quality by reducing the number of Zn interstitials and formed electron traps, which weakened the field emission and hence led to an *E*_on_ of 9 to 22.5 V μm^−1^.^12^ Despite the fact that doping of elements like Ga,^13^ Al,^14^ Mg,^15^ C,^16^ In^17^*etc.* resulted in a favorable alteration of the electronic properties of ZnO which might have assisted in the lower possible *E*_on_ values of 2.4, 2.8, 5.99, 18 and 193 V μm^−1^, respectively, for field emission, one cannot neglect that these values are defined at a lower current density ranging from 0.1 to 1 μA cm^−2^.

The modification of critical surface bond length in the nano-regime can tailor the ZnO nanostructure morphologies of the pyramid-, pencil-, rod-, wire-, *etc.*, forms.^18^ However, metals were employed as catalysts in the growth process for control over the random alignment and density of the structures, which unfavorably tailored the field emission properties. The density controlled growth of ZnO nanopillars using self-assembled Ag nano-islands/layers resulted in an *E*_on_ of 2.39 V μm^−1^.^19^ Catalysts guided the vertical alignment of the ZnO nanowires on an insulating substrate such as sapphire which limited their application in photonic/electronic devices like field emitters.^20^ On the other hand, it has been demonstrated that the needle morphological forms can emit electrons more easily.^21^ Many growth methods have been utilized to explore various 1D morphologies^4^ but the very few which are known to provide tip features are cursed with post-treatments such as annealing or *in situ* heating. The air annealed tip-morphology of ZnO nanorods exhibited an *E*_on_ of 3.5 V μm^−1^ owing to its large rod-body diameter and shortened tips.^21^*C*-Axis oriented ZnO nanocones *in situ* heated at 580 °C in an O_2_ atmosphere to grow on a Si substrate had an *E*_on_ of 2.57 V μm^−1^ defined at a very low current density of 0.1 μA cm^−2^.^22^ Zhao *et al.*^23^ thermally annealed ZnO nanorods in oxygen, air and NH_3_ to improve the *E*_on_ (at 0.1 μA cm^−2^) from 8.8 V μm^−1^ to 4.1 V μm^−1^. Ghosh *et al.*^24^ observed an enhancement in FE performance after capping the tips of randomly oriented and highly oxygen defective ZnO nanostructures with metal nanoparticles despite their larger values of work function (*i.e.* 5.04–4.7 eV). Therefore, for promising field emission performance, efforts on size reduction, uniform morphology, sharp tip features and periodic growth of pristine ZnO nanowires appears to be of scientific and technological importance.

In this work, we present the synthesis of large-area arrays of randomly oriented spitzer shaped truncated/pointed ZnO nanowires grown periodically like Christmas trees using hydrothermal methods as excellent field emitters. The influence of the spitzer shaped tip morphologies of the 1D ZnO nanowires on field electron emission properties was studied methodically. The surface morphological features, and chemical and electronic structure of pristine ZnO nanowires were revealed using field-emission scanning electron microscopy (FESEM) and X-ray photoelectron spectroscopy (XPS). The FE behaviors of pristine 1D ZnO nanowires were studied at optimized anode–cathode separation and it was observed that at a separation of 2000 μm the 1D spitzer shaped hexagonal ZnO nanowires exhibited excellent FE properties.

## Experimental

Large area arrays of ZnO nanowires were synthesized on ITO coated glass substrates *via* a hydrothermal approach. Zinc acetate dihydrate (C_4_H_6_O_4_Zn·2H_2_O, 98%, Sigma Aldrich) and sodium peroxide (Na_2_O_2_, 97%, Sigma Aldrich) at 30 mM L^−1^ and 100 mM L^−1^ concentrations, respectively, were mixed to form a 1 : 1 solution. This solution was stirred at room temperature for 30 min and then transferred to an autoclave containing well aligned ITO coated glass substrates. The hydrothermal reaction was carried out at 85 °C for 12 h to grow 1D ZnO nanowires over the ITO coated glass substrate. After that, the surface morphology of the pristine ZnO nanowires was confirmed using field emission scanning electron microscopy (FESEM, Carl Zeiss, Merlin 6073). The chemical states of the ZnO nanowires were analyzed using an X-ray photoelectron spectrometer (XPS, Thermo Scientific Inc. K_α_) with a microfocus monochromated Al K_α_ X-ray. The valence band spectra (VBS) were measured using an Omicron energy analyzer (EA-125, Germany) with an angle incidence photo-emission spectroscopy (AIPES) beamline on an Indus-1 synchrotron source at RRCAT, Indore, India. The FE studies of the pristine ZnO nanowires were carried out in a vacuum chamber maintained at a base pressure of ∼7.5 × 10^−9^ Torr. The anode semi-transparent phosphor screen was maintained at various distances of 1500, 2000 and 2500 μm from the pristine ZnO nanowires (≡ZnO/ITO emitter). Samples were preconditioned at a voltage of ∼3 kV for 30 min to avoid the influence of contamination and loosely bound nanowires in the field emission. The field emission current (*I*) was measured with an electrometer (Keithley 6514) at a direct current (dc) voltage (*V*) applied using a high-voltage dc power supply (0–40 kV, Spellman). The long-term stability of the field emission current was recorded for the ZnO/ITO emitters.

## Results and discussion

**Fig. 1:**
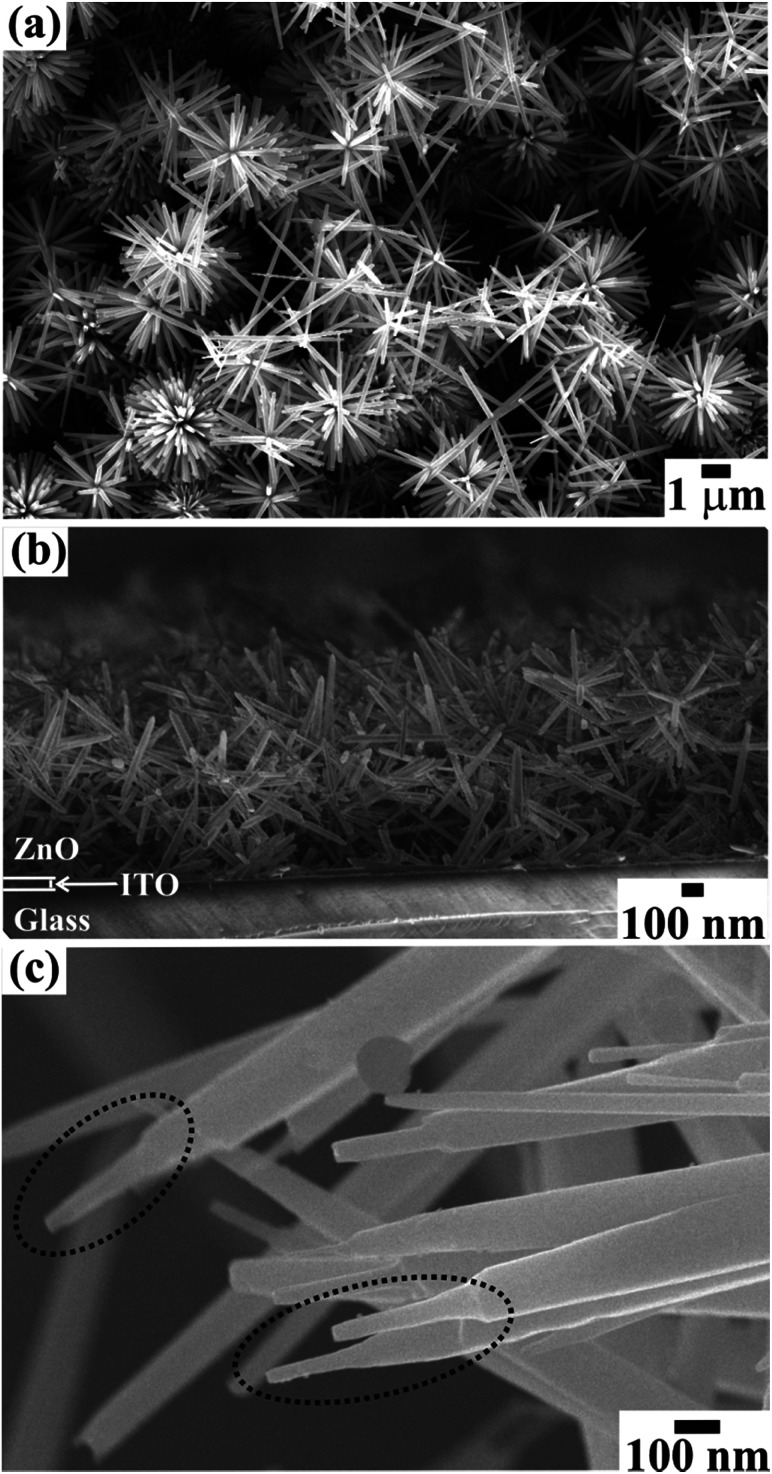
FESEM images showing the (a) top view and (b) side view of a large area array of ZnO nanowires with (c) spitzer shaped morphologies grown on ITO coated glass substrate.

**Fig. 2:**
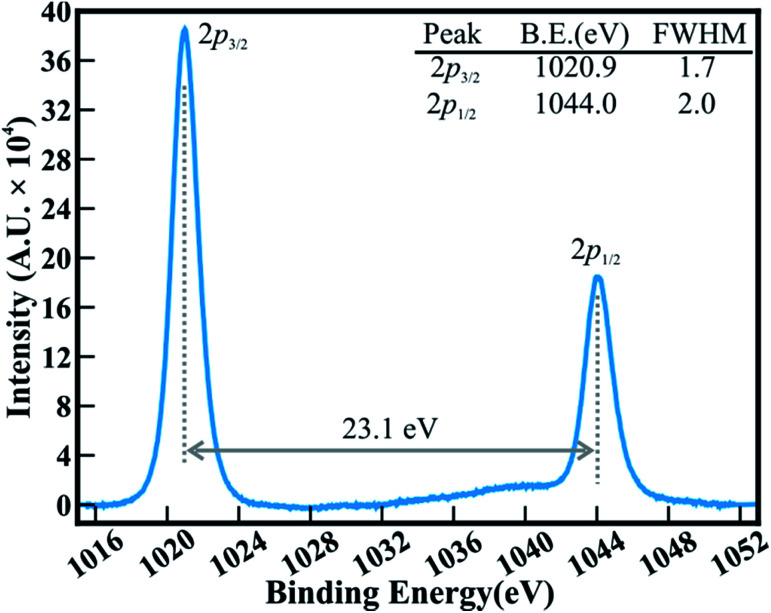
High-resolution XPS spectra of the Zn(2p) core levels of spitzer shaped ZnO nanowires.

The FESEM images in Fig. 1 show the surface morphology of large area arrays of ZnO nanowires grown over ITO coated conducting glass substrates. The hexagonal ZnO nanowires are confined to a limited range of diameter (<180 nm). The well-separated nanowires with clearly visible textural boundaries were of an average body diameter of ∼120 nm and were ∼5–6 μm long (Fig. 1(a)). These distinct ZnO nanowires were well arranged in the form of Christmas trees which appeared like a forest of well separated and periodically arranged trees, (Fig. 1(a)), to deliver highly porous thin films of thickness ∼1300 nm (Fig. 1(b)). Close examination of the ZnO nanowire array revealed the curtailing of the hexagonal facets at its tip, which resulted in the formation of spitzer shaped truncated/pointed tips (Fig. 1(c)). The spitzer shaped ZnO nanowires with truncated tips at the top of the trees had diameters less than ∼30 nm (Fig. 1(c)). The spitzer shaped truncated tip construction of ZnO nanowires is expected to govern the enhanced FE behaviors. The electronic structure and chemical properties of spitzer shaped ZnO nanowires were confirmed by XPS investigations. Fig. 2 shows the high-resolution XPS spectra of the Zn(2p) core level of the ZnO nanowires. The clearly observable double peak feature of Zn(2p_3/2_) and Zn(2p_1/2_) located at a binding energy of 1020.9 (±0.1) and 1044.0 (±0.1) eV, respectively, represents the core level of Zn^2+^ cations.^5,25^ The estimated energy separation of 23.1 eV, assigned to ZnO and not metallic Zn,^26^ was maintained between the Zn(2p) core levels of the spitzer shaped nanowires.

**Fig. 3:**
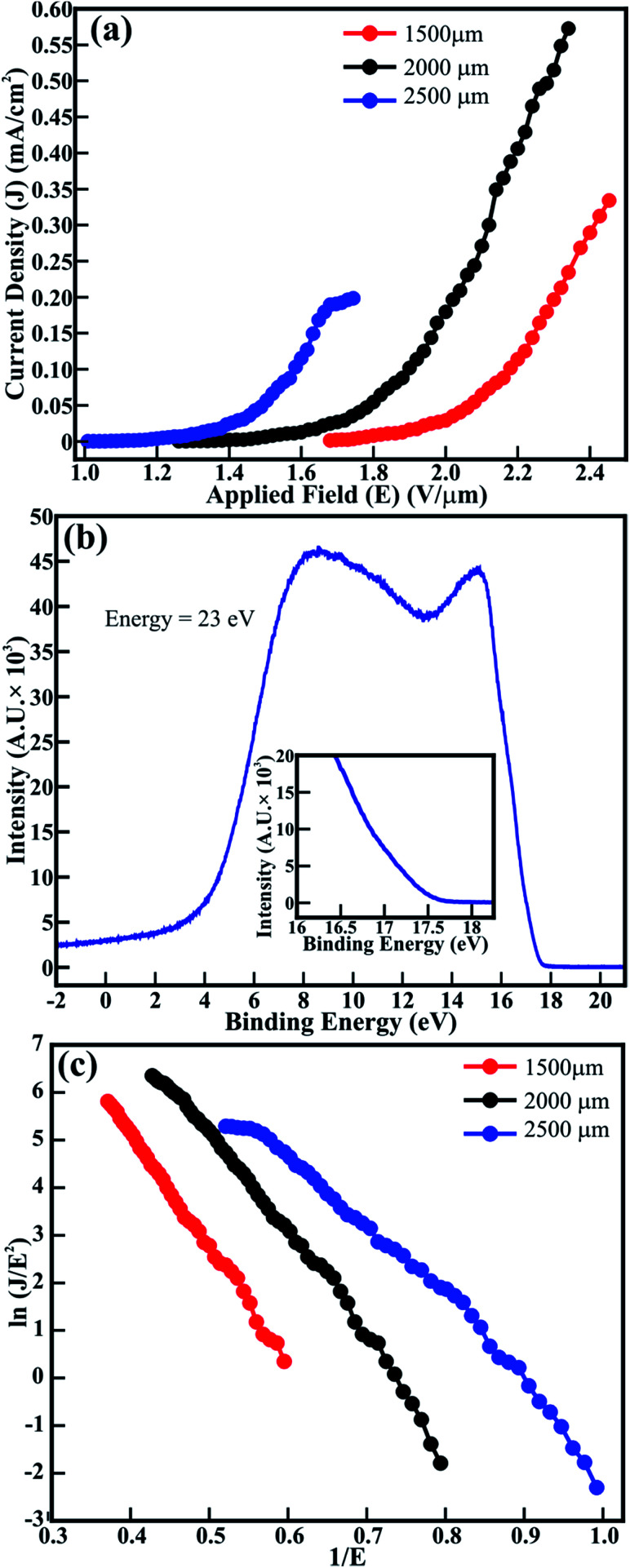
Field emission (a) *J*–*E* curves and (c) F–N plots obtained from the *J*–*E* curves, and (b) UPS valence band spectra measured for spitzer shaped ZnO nanowires. The inset in (b) shows the magnified valence band spectra at the higher binding energy.

The FE measurements of the spitzer shaped 1D ZnO nanowires (≡1D ZnO/ITO) were performed in the planer diode configuration. The emitting device with an emission area of ∼0.30 cm^2^ was maintained with anode–cathode separations of 1500, 2000 and 2500 μm. The applied electric field (*E*) dependent variation in the electron emission current density (*J*) (*i.e. J*–*E* plot) of the ZnO/ITO emitters is shown in Fig. 3(a). Although spitzer shaped ZnO nanowires are periodically arranged in the form of trees, their random orientation leads to the applied electric field (*E* = *V*/*d*_sep_) being treated as the average field and not the uniform field between the electrodes separated by the distance *d*_sep_. The spitzer shaped ZnO nanowires (≡ZnO/ITO) were subjected to electron field emission at separations of 1500, 2000 and 2500 μm to confirm the optimized field emission behavior. The large emission current density of 572 μA cm^−2^, lower threshold field (*E*_thr_) of 1.9 V μm^−1^ and lower *E*_on_ of 1.56 V μm^−1^ were observed at 2000 μm. However, the lowest *E*_on_ (1.16 V μm^−1^) was observed at the separation of 2500 μm with the emission current density decreased significantly to 198 μA cm^−2^. The *E*_on_ observed for these spitzer shaped truncated tip ZnO nanowire arrays is much lower than that reported for ZnO nanorods grown on Si substrates using PLD (*i.e.* 2 V μm^−1^),^6^ ZnO nanopillers grown by vapor transport deposition (*i.e.* 3.15 V μm^−1^),^27^ ZnO nanorods and nanodisk networks (*i.e.* 4.8 and 2.6 V μm^−1^, respectively, reported at 1 μA cm^−2^),^7^ ZnO nanoneedles (*i.e.* 2.4 V μm^−1^) and bottle-like nanorods (*i.e.* 4.6 V μm^−1^) fabricated using vapor phase growth,^8^ ZnO agave-like (*i.e.* 2.4 V μm^−1^) and pencil-like (*i.e.* 3.7 V μm^−1^) nanostructures grown on amorphous carbon,^9^ nitrogen implanted ZnO nanowires (*i.e.* 2.4 V μm^−1^ at a current density of 0.1 μA cm^−2^),^11^ metal (Ag/Pt/Au) loaded ZnO nanorods (*i.e.* 1.9 and 2.6 V μm^−1^, respectively),^28^ CuO nanoplates (*i.e.*, 6.7 V μm^−1^),^3^ ZnO nanotetrapods screen-printed on carbon nanofiber buffered Ag (*i.e.* 6.7 V μm^−1^ defined at 0.1 μA cm^−2^)^29^ and brookite TiO_2_.^2,30^*C*-Axis oriented ZnO nanocones were expected to deliver better field emission because of the tapered cone-like morphology, nevertheless, the *E*_on_ obtained at a very low current density of 0.1 μA cm^−2^ was restricted to 2.57 V μm^−1^ which might be due to the very low areal density of ZnO nanocones.^22^ Dense morphology reported as hexagonal flower-like ZnO nanowhiskers delivered an *E*_on_ of 2.2 V μm^−1^ (at a current density of 0.1 μA cm^−2^) which might be due to the diameter of the whiskers being limited to 300 nm.^31^ The n-type nitrogen^32^ or H-plasma^33^ treated ZnO nanowires were not successful at improving the *E*_on_ beyond 2.1 V μm^−1^. Furthermore, Sugavaneshwar *et al.*^34^ have reported an enhancement in the field emission of branched ZnO nanostructures compared to that of simpler nanostructures such as nanowires and nanorods, *etc.*, but the actual values of *E*_on_ were not stated. Although Chang *et al.*^27^ have reported an enhancement in the FE properties of ZnO nanopillars after decorating Au nanoparticles along the surface, the minimum *E*_on_ of the ZnO nanopillars which was limited to 3.15 V μm^−1^ was further reduced to 2.65 V μm^−1^ (after Au decoration) owing to the larger diameter and flat top of the ZnO nanopillars (*i.e.* ∼200 nm). Additionally, the selective patterning of ZnO nanorods achieved an *E*_on_ of 2.85 V μm^−1^.^35^ The possible reasons behind such higher turn-on values are the flat tips, nonuniform morphologies, uneven distribution and larger diameter of the 1D ZnO structures. Moreover, the emission current density of 572 μA cm^−2^ attained at a lower applied field of 2.34 V μm^−1^ for ZnO nanowires (≡ZnO/ITO) is reasonably higher than that reported for pristine and Al (*i.e.* ∼4 μA cm^−2^ and ∼3 μA cm^−2^, respectively),^14^ C (*i.e.* 16 μA cm^−2^)^16^ and In (*i.e.* 1.5 μA cm^−2^)-doped ZnO nanostructures.^17^ In contrast, pristine ZnO and Mg-doped ZnO nanostructures have drawn slightly better emission currents (*i.e.* 0.8 to 3.2 mA cm^−2^) at the much higher applied field of 9.2 V μm^−1^.^15^

Reduction of the work function enhances FE properties. Therefore, ultraviolet photoelectron spectroscopy (UPS) was utilized to estimate the work function of ZnO/ITO emitters. The UPS spectra recorded for a ZnO nanowire array at an energy of 23 eV is shown in Fig. 3(b). Two distinct peaks in the VBS of ZnO nanowires located at higher and lower binding energies are assigned to the hybridization of the O(2p) and Zn(4s) orbitals, and nonbonding O(2p) orbitals respectively.^36^ Moreover, the work functions of ZnO emitters are estimated from the equation^37^

1 *Φ*_ZnO_ = *hω* − |*E*_sec_ − *E*_FE_|

where *hω* is the energy of the source utilized (≡23 eV), *E*_sec_ is the onset of the secondary emission and *E*_FE_ is the Fermi edge. The spitzer shaped truncated tip appearance of the ZnO nanowires resulted in a lower work-function of 4.9 eV (*i.e. Φ*_ZnO_) than that of reported ZnO nanostructures such as nanorods and nanowires,^10,14^ oxygen plasma treated ZnO (*i.e.* 5.5 eV),^33^ Ag decorated ZnO nanorods (*i.e.* 4.7 eV),^10^ and Au faceted oxygen-deficient ZnO nanostructures.^24^ In the present case, the reduction in work-function is thought to originate from the spitzer shaped truncated tip morphology which might have helped enhance the FE behavior of the ZnO nanowires.

A modified Fowler–Nordheim (F–N) equation (*i.e.*eqn (2)) is used to substantiate the variation in the emission current density of ZnO/ITO emitters subject to the applied field,2
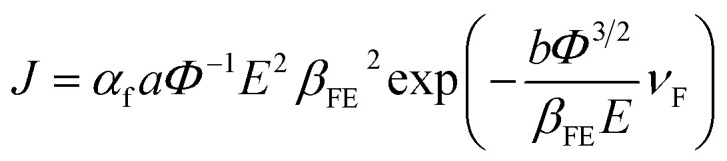
where *J* is the average FE current density of the device, *α*_f_ is a macroscopic pre-exponential correction factor, *a* and *b* are constants (*a* = 1.54 × 10^−6^ A eV V^−2^ and *b* = 6.83089 × 10^3^ eV^−3/2^ V μm^−1^), *Φ* is the work function of the emitter (*i.e. Φ*_ZnO_ = 4.9 eV), *E* is the average applied electric field, *β*_FE_ is the local electric field enhancement factor and *ν*_F_ is the correction factor also known as the specific value of the principal Schottky–Nordheim barrier function, *ν*. Due to the random alignment of ZnO nanowires confirmed from FESEM images (Fig. 1), the emission surface of the ZnO/ITO emitters is treated as rough.

Therefore, applied and local electric fields at emission sites (*i.e.* ZnO) differ from each other, and their ratio is identified as *β*_FE_. A plot of ln{*J*/*E*^2^} *versus* (1/*E*), accepted as a F–N plot, is illustrated using eqn (2), and the field enhancement factor (*β*_FE_) is estimated from equation3
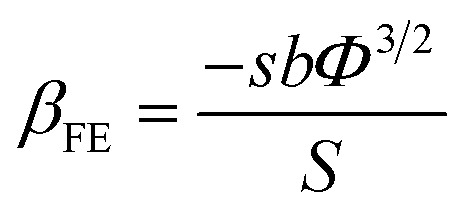
where *s* (=0.95), the slope correction factor for the Schottky–Nordheim barrier, is 1 in the present case, for simplicity.

The F–N plots for pristine ZnO/ITO emitters determined at various anode–cathode separations are shown in Fig. 3(c). The distinct F–N plots are ascribed to the well-defined band alignment of the nanowire morphology of ZnO. The optimized anode–cathode separations in pristine ZnO/ITO emitters have tailored the values of *β*_FE_. The *β*_FE_ values estimated for ZnO/ITO emitters at the anode–cathode separations of 1500, 2000 and 2500 μm are 3089, 3924 and 4760, respectively. These estimated values of *β*_FE_ for ZnO/ITO emitters are more significant than the reported values of ZnO nanostructure arrays with nanoneedle, nanocavity and bottle shaped morphologies,^8^ hierarchical and pencil-like ZnO nanostructures self-assembled on amorphous carbon,^9^ ZnO branched nanostructures,^34^ density controlled ZnO nanopillar arrays,^19^ tapered ZnO nanorods grown on Fe and Cu electrodes,^38^ metal doped ZnO nanowires,^14,15,17^ composites of carbon–ZnO,^39^ and MoS_2_@ZnO nano-heterojunctions.^40^ Bae *et al.*^22^ revealed a field enhancement factor of 2216 for ZnO nanocones, which was not improved after tailoring the density of the nanocones in the emission area. Sugavaneshwar *et al.*^34^ tailored vapor phase transport to synthesize ZnO nanostructures in the form of wires and branches, but their larger diameter restricted the *β*_FE_ values in the range of 1129 to 3985. Although Naik *et al.*^41^ and Jing *et al.*^42^ have reported larger values of *β*_FE_ for ZnO nanosheets and nanotowers, respectively, the orthodoxy test known to authenticate such values was not performed to support this. The expediency of the FE measurements and *β*_FE_ of the ZnO/ITO emitters was confirmed by performing an orthodoxy test utilizing the spreadsheet provided by Forbes in ref. 43. The scaled-barrier-field (*f*) values estimated for all of the cathode–anode separations in ZnO/ITO emitters are given in Table 1.

**Table 1:** Scaled-barrier-field (*f*) values estimated from F–N plots for ZnO nanowire (*i.e.* ZnO/ITO) emitters using the spreadsheet from ref. 43

Materials	Separation (μm)	*f*_low_	*f*_high_	Orthodoxy test result
ZnO nanowires	1500	0.29	0.47	Pass
	2000	0.24	0.44	Pass
	2500	0.26	0.50	Pass

The emission situation is orthodox throughout all of the cathode–anode separations of Zn/ITO emitters for the lower (*f*_low_) as well as the higher (*f*_high_) scaled-barrier-field (*f*) values. The hexagonal ZnO nanowires with clearly visible textural boundaries revealed reasonable emission behavior for all of the maintained anode–cathode separations. The unique morphological features of the ZnO nanowires, such as hexagonal morphology, individual dispersion, spitzer shaped truncated tips and very sharp pointed tips have resulted in low *E*_on_ values and large values of *β*_FE_ for the ZnO/ITO emitters.

**Fig. 4:**
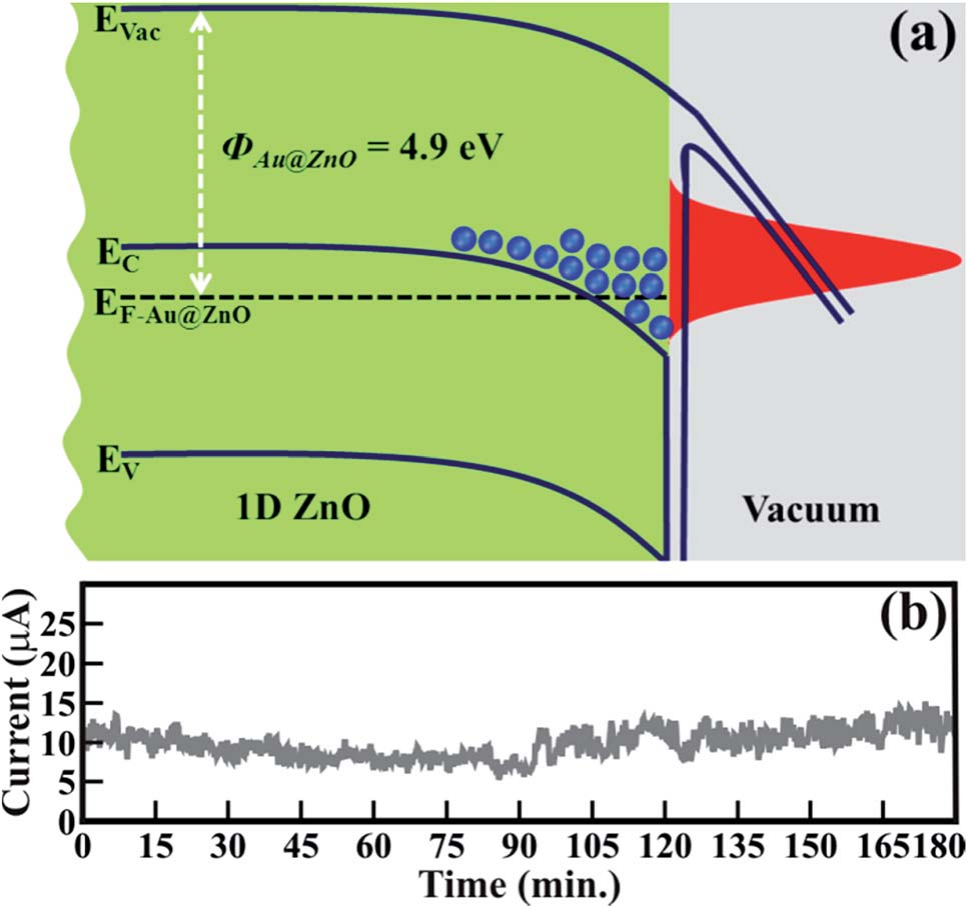
(a) Schematic band alignment of pristine spitzer shaped ZnO nanowires and (b) field emission current stability (*I*–*t*) plot of 1D ZnO nanowires.

This emission behavior can be described in more detail by considering the band alignment of ZnO (Fig. 4(a)). In the present case, owing to unique morphological features, the work function of the ZnO nanowires (*i.e.* 4.9 eV) has been reduced compared to that of reported values (*i.e.* 5.5 to 5.2 eV).^6–9^ The reduced *Φ*_ZnO_ provides a significantly smaller barrier for the emission of an electron. Therefore, enhancement in FE behavior is expected along with lower *E*_on_ values and higher values of *β*_FE_. In the case of ZnO/ITO emitters, the electrons from the conduction band or its nearest states contribute to field emission. Moreover, at an applied electric field, energy band bending generates energy well at a depleted region where a large number of electrons accumulate and are then abruptly emitted in larger quantities due to a relatively lower *Φ*_ZnO_ (Fig. 4(a)).

Stable field electron emission (*i.e.* current) is one of the prerequisites for utilizing materials for the fabrication of FE displays and related applications. Fig. 4(b) shows the FE stability of ZnO/ITO emitters. The emission current (*I*) at an applied voltage of 10 μA, assigned as *E*_on_, was considered to confirm the stability of ZnO/ITO emitters. A negligible amount of current fluctuation (*i.e.* an average of ±5%) was observed even after continuous emission for 180 min. These spitzer shaped 1D ZnO nanowires exhibited very stable and improved electron emission than that of gold nanoparticle decorated ZnO nanopillars,^27^ monolayer graphene supported by well-aligned ZnO nanowire arrays grown on Si substrates,^44^ seed layer assisted ZnO nanorods,^45^ ZnO nanowires derived after annealing gold deposited Zn substrate at 400 °C,^46^ and ZnO multipods, submicron wires and spherical structures obtained by vapour deposition.^47^ The exclusive participation of the sharp tips of the ZnO nanowires as emitters conceivably enhanced the emission ability.

## Conclusions

In conclusion, the large area array of stoichiometric and individual dispersed 1D hexagonal ZnO nanowires of spitzer shaped, truncated and very sharp pointed tips synthesized on ITO coated glass substrates resulted in a smaller work-function of 4.9 eV which consequently delivered a significantly smaller *E*_on_ of 1.56 V μm^−1^ and stable electron emission (*i.e.* average current fluctuations of ±5%). These spitzer shaped ZnO nanowires have potential for utilization in vacuum based micro/nano-devices such as flat-panel displays and intense point electron sources. Moreover, the ZnO nanowires have capabilities to further reduce the work-function and improve electron emission, as well to expand other functionalities for various applications, after the controlled design of nano-hetero-architectures with metals or highly conducting materials.

## Conflicts of interest

There are no conflicts to declare.

## Supplementary Material
